# AHP6 Inhibits Cytokinin Signaling to Regulate the Orientation of Pericycle Cell Division during Lateral Root Initiation

**DOI:** 10.1371/journal.pone.0056370

**Published:** 2013-02-14

**Authors:** Sofia Moreira, Anthony Bishopp, Helena Carvalho, Ana Campilho

**Affiliations:** 1 Institute for Molecular and Cell Biology (IBMC), University of Porto, Porto, Portugal; 2 Institute of Biotechnology, University of Helsinki, Helsinki, Finland; Iwate University, Japan

## Abstract

In *Arabidopsis thaliana*, lateral roots (LRs) initiate from anticlinal cell divisions of pericycle founder cells. The formation of LR primordia is regulated antagonistically by the phytohormones cytokinin and auxin. It has previously been shown that cytokinin has an inhibitory effect on the patterning events occurring during LR formation. However, the molecular players involved in cytokinin repression are still unknown. In a similar manner to protoxylem formation in *Arabidopsis* roots, in which AHP6 (ARABIDOPSIS HISTIDINE PHOSPHOTRANSFER PROTEIN 6) acts as a cytokinin inhibitor, we reveal that AHP6 also functions as a cytokinin repressor during early stages of LR development. We show that *AHP6* is expressed at different developmental stages during LR formation and is required for the correct orientation of cell divisions at the onset of LR development. Moreover, we demonstrate that *AHP6* influences the localization of the auxin efflux carrier PIN1, which is necessary for patterning the LR primordia. In summary, we show that the inhibition of cytokinin signaling through AHP6 is required to establish the correct pattern during LR initiation.

## Introduction

Plants have the capacity to form new organs, such as lateral roots, leaves, and flowers during postembryonic development. Organ primordia develop from populations of founder cell into organs through the coordinated process of cell division and differentiation. Lateral roots (LRs) originate from a small number of differentiated pericycle cells adjacent to xylem poles, called pericycle founder cells (reviewed in [1]). These founder cells undergo a defined program of oriented cell divisions and expansion to initiate, pattern and allow the emergence of the LR primordia. This is followed by the activation of a new meristem and elongation of the new LR (reviewed in [1]). The formation of LR primordia is antagonistically regulated by the phytohormones auxin and cytokinin (CK). It has been shown that establishing an auxin gradient with its maximum at the root tip is essential for proper LR patterning, and this process is dependent on the polar transport of auxin mediated by auxin efflux carriers (such as PIN1) [2]. CKs are negative regulators of LR formation. Plants with reduced levels of CK or CK signaling exhibit enhanced root branching [3], [4]. Furthermore, it was shown that CKs act directly on pericycle founder cells to disrupt LR initiation and patterning [5]. This implies that CK interferes with very early patterning events. The current consensus is that CK disrupts LR patterning by interfering with the expression of auxin efflux carrier genes, and therefore disturbing the formation of an auxin gradient [5]. Recently, it has been shown that during LR development CK regulates endocytic recycling of the auxin efflux carrier PIN1 by redirecting it for lytic degradation in vacuoles [6]. However, the molecular components involved in the repression of CK signaling in LRs are still unknown and consequently the molecular mechanisms through which CK and auxin interact to produce this specific developmental output are unclear.

A mechanism for cytokinin repression has been identified during vascular patterning. Perception of CK and transmission of that signal occurs through a two-component phosphorelay signaling system in which histidine phosphotransfer proteins transfer the phosphoryl group from membrane-bound histidine kinases receptors to the nuclear CK response regulators (RR), which ultimately activate transcription of downstream targets [7]. AHP6 is a “pseudo- histidine phosphotransfer protein” that contains a mutation in the conserved histidine residue required to accept the incoming phosphoryl group from the receptors. *AHP6* is expressed in specific cell files where it inhibits CK signaling and allows the specification of protoxylem cell identity [8]. During vascular development, a mutually inhibitory interaction between CK and auxin determines the position of the xylem axis and specifies a bisymmetric pattern of distinct domains of auxin and cytokinin signaling output in the root vascular cylinder [9]. In this mechanism an auxin response maximum in the xylem axis [9], [10] promotes the expression of *AHP6* as a primary auxin response gene and this inhibits CK signaling at the protoxylem position. High cytokinin signaling affects the expression and subcellular localization of various PIN proteins that promote the radial transport of auxin [9].

In this study, we report that AHP6 acts as an inhibitor of cytokinin signaling that is necessary to initiate patterning of the lateral root and we propose that it acts by modulating the localization of the auxin efflux carrier, PIN1, and through this affects auxin distribution.

## Results

### *AHP6* is expressed early during lateral root development

**Figure 1:**
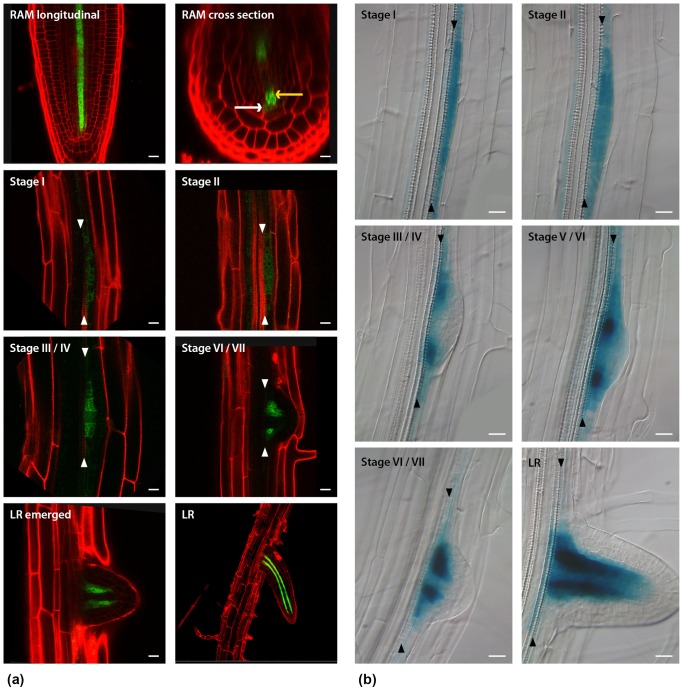
*AHP6* is expressed from the initial stages of lateral root development. a) *AHP6::GFP* expression in the root apical meristem (RAM) and throughout different stages of lateral root (LR) development; the longitudinal and cross section images were obtained using the horizontal xy-section and vertical xz-section of confocal scan-mode, respectively. b) *AHP6::GUS* expression at different LR developmental stages: from stage I to an emerged LR. Yellow arrow: protoxylem cell; white arrow: protoxylem-associated pericycle cell. Arrowheads: Xylem cell files. Bars: 10 µm.

To investigate if AHP6 has a role as a cytokinin inhibitor during lateral root development, we firstly characterized *AHP6* expression along the primary root using both GFP and GUS transcriptional fusions. As previously described, *AHP6* is expressed at the root apical meristem (RAM) in the protoxylem and the protoxylem-associated pericycle cell files ([8] and Figure 1a – RAM). As cells exit the meristem and enter the elongation zone, expression of *AHP6* is reduced and eventually switched off. However, we observed additional zones of *AHP6* expression during early stages of lateral root development (Figure 1a and 1b). Lateral root organogenesis is defined by a specific program of cell divisions and anatomical changes, which have been divided into 8 stages [11]. At stages I and II, *AHP6* is ubiquitously expressed in all cells of the lateral root primordia (Figure 1a and 1b). From stage III onwards *AHP6* expression becomes restricted to two domains at the margin of the primordia where the vascular tissues will form between the main and the lateral root (Figure 1a and 1b). In the emerged lateral root, *AHP6* is expressed in two poles within the newly formed vascular cylinder and in the lateral root meristem (Figure 1a and 1b). Our data reveals that *AHP6* is expressed at all stages of lateral root development, including very early stages. This introduces the possibility that *AHP6* may have a role in lateral root initiation.

### *AHP6* is required to orient the first cell divisions of lateral root formation

To test whether *AHP6* functions during lateral root development, we analyzed different aspects of this developmental process in wild-type (WT) plants and *ahp6* mutants. We initially quantified the density of lateral roots and lateral root primordia in 10 dpg WT seedlings and in two *ahp6* mutant alleles, *ahp6-1* and *ahp6-3*. We found that these values were similar between WT and both alleles of *ahp6* (Figure S1a and S1b). Next, we quantified primary root growth and the distribution of the different stages of lateral root primordia along 10 dpg primary root in WT, *ahp6-1* and *ahp6-3* seedlings. There were no significant differences for root growth between WT and the two mutant alleles (Figure S1c) as well as for the distribution of lateral root primordia (Figure S1d). We could detect a tendency for a decrease in the number of emerged lateral roots in *ahp6* when compared to WT (Figure S1d - Emerged roots (E)). AHP6 has previously been shown to affect the activity of the shoot apical meristem [12]. As the formation of the first true leaves provides a significant auxin input into the root that promotes the emergence of lateral roots [13], we tested whether the reduced number of emerged lateral roots in *ahp6* could be due to changes in shoot architecture. We compared the timing at which the first true leaves were visible in wild-type plants and *ahp6* mutants but were unable to observe a statistically significant difference. The first leaves were present 6 days after germination in 94.3% of wild-type seedlings (n = 121) and 96.3% of *ahp6* mutants (n = 164). This suggests that the shoots of *ahp6* mutants and wild-type seedlings are likely to be of a comparative developmental stage, and that it is unlikely that any differences in the frequency of emerged lateral roots would be primarily due to deviations in auxin input caused by the initiation of the first true leaves. This gave us the confidence to explore a root-specific role for AHP6 during lateral root formation.

**Figure 2:**
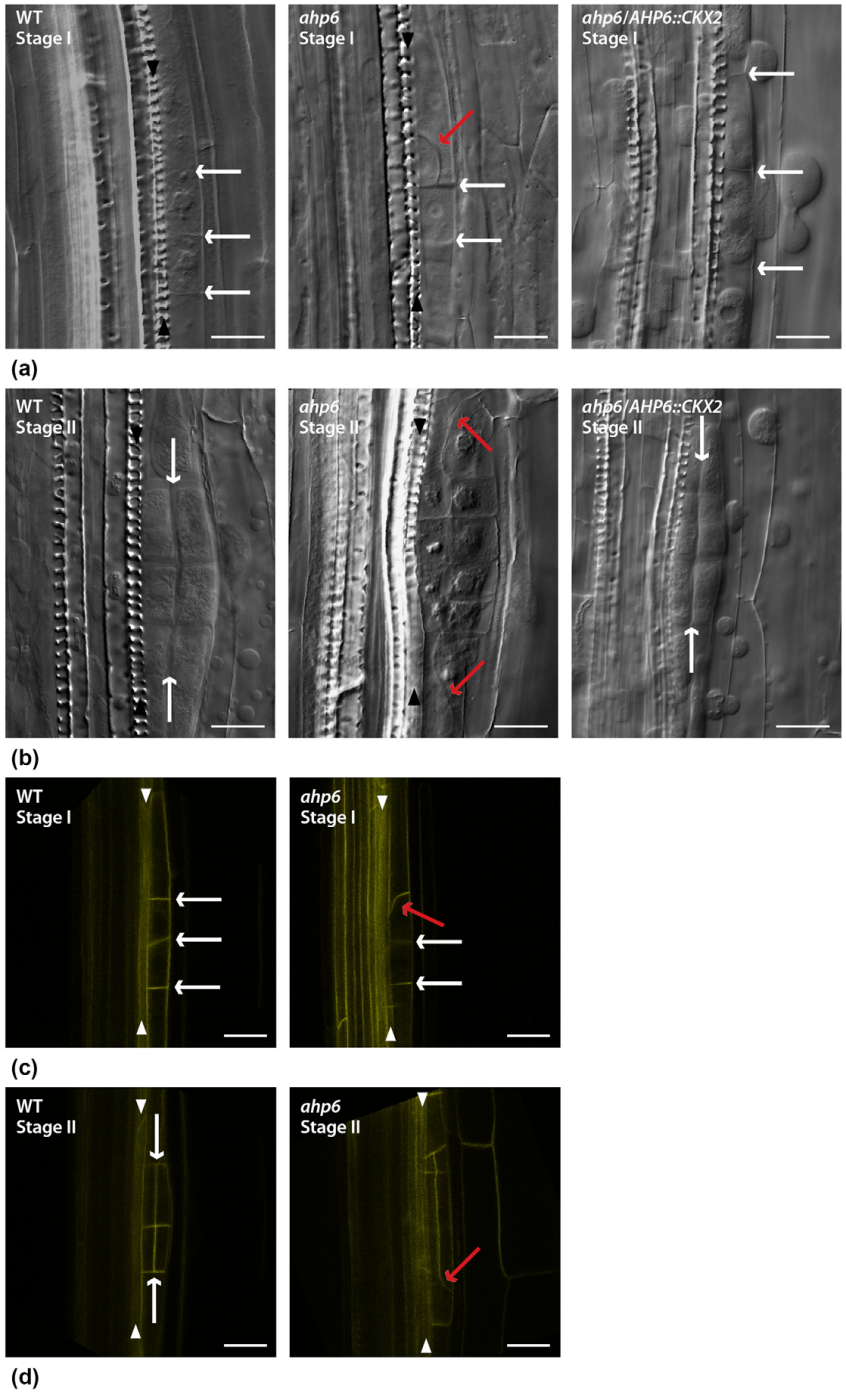
Abnormal cell division orientation of stage I and stage II *ahp6* lateral root (LR) primordia. a) Differential Interference Contrast (DIC) images of WT anticlinal pericycle founder cell divisions at stage I (white arrows) and a defective cell division (red arrow) in *ahp6* at the same LR developmental stage; pericycle founder cell divided in the normal anticlinal orientation in *ahp6/AHP6::CKX2* (white arrows). b) DIC images of WT and periclinal cell divisions of stage II (white arrows) and defective cell divisions (red arrows) in *ahp6* at the same LR developmental stage; normal periclinal cell divisions in *ahp6*/*AHP6::CKX2* (white arrows). c) AUX1-YFP as fluorescent marker to label the plasma membranes and show a WT stage I LR primordia anticlinal cell divisions (white arrows) and an abnormal cell division at stage I *ahp6* primordia (red arrow). d) AUX1-YFP as fluorescent marker to label the plasma membranes and show a WT stage II LR primordia periclinal cell divisions (white arrows) and abnormal cell division orientation at stage II *ahp6* primordia (red arrows). Arrowheads: Xylem cell files. Bars: 20 µm.

**Figure 3:**
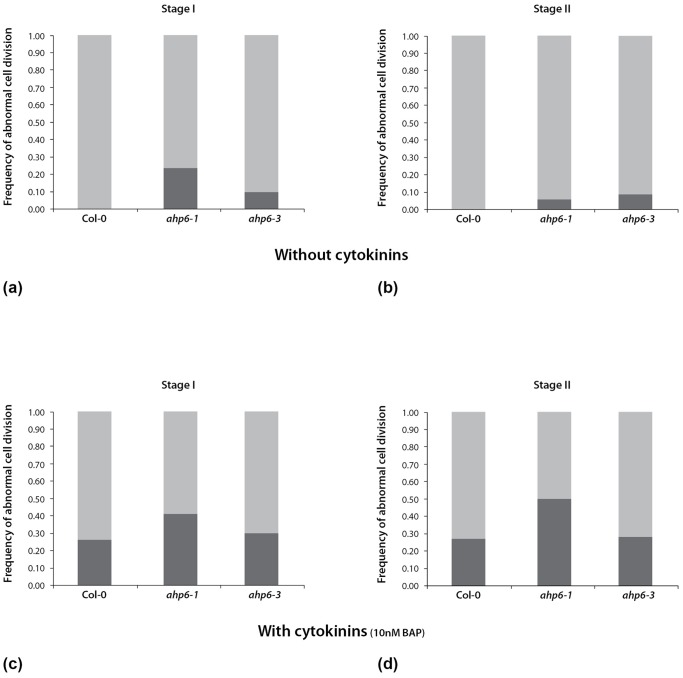
Frequency of abnormal cell divisions at stage I and stage II lateral root (LR) primordia. a) At stage I, wild-type (WT) LR primordia (n = 40) show an invariant pattern where all cell divisions occur in an anticlinal orientation. In contrast, in LR primordia of *ahp6* mutants at the same developmental stage show abnormalities in the plane of cell division: ≈25% for *ahp6-1* (n = 37) and 10% for *ahp6-3* (n = 48) LR primordia. b) At stage II, a similar invariant pattern of cell division was observed in the WT LR primordia (n = 61) with all cell divisions occurring in a periclinal orientation, whereas abnormally orientated cell divisions occurred in ≈5% for *ahp6-1* (n = 72) and 10% for *ahp6-3* (n = 55) LR primordia. This is data combined from three independent experiments with a total number of 58 WT roots, 84 *ahp6-1* roots and 75 *ahp6-3* roots. c) When grown with 10 nM cytokinin, about 25% of stage I WT LR primordia (n = 35) show abnormal cell divisions. There is also an additive increase in the number of abnormal cell divisions in CK treated *ahp6-1* mutants with ≈40% of stage I LR primordia (n = 41) showing abnormal periclinal cell divisions. This effect is smaller in *ahp6-3* where ≈30% of stage I LR primordia (n = 37) show aberrant cell divisions. d) When grown with 10 nM cytokinin, there is about 25% increase in the number of WT stage II LR primordia (n = 48) with abnormal orientation of cell divisions. The frequency of cell divisions with aberrant orientations is also increased in *ahp6* stage II LR primordia: ≈50% for *ahp6-1* (n = 46) and ≈30% for *ahp6-3* (n = 53). This is data combined from two independent experiments with a total number of 51 WT roots, 58 *ahp6-1* roots and 56 *ahp6-3* roots.

Interestingly, we also observed defects in the orientation of cell divisions in the early stages of lateral root primordia formation in *ahp6*. In WT, lateral roots are initiated through invariant anticlinal cell divisions of the pericycle founder cells (Figure 2a and 2c - WT stage I), followed by a subsequent round of periclinal cell divisions (Figure 2b and 2d - WT stage II). In *ahp6* mutants, we observed abnormal tangential or oblique cell divisions in the pericycle founder cells at stage I (Figure 2a and 2c - *ahp6* stage I) and stage II (Figure 2b and 2d - *ahp6* stage II). We quantified the relative frequency with which the abnormal pericycle cell divisions occurred in stage I and II for WT and the two alleles of *ahp6*. Later stages of lateral root development were not included in the analysis due to a higher variation in the orientation of cell divisions in WT. Abnormal pericycle cell divisions are totally absent in WT stage I and stage II but occur in 5% to 25% LRP in both *ahp6* mutant alleles (Figure 3a and 3b) (see also Table S1). Despite the fact that this phenotype is subtle and incompletely penetrant, we were able to observe this repeatedly in three independent replicated experiments.

Our data show that the *ahp6* mutant displays defects in the pericycle founder cell divisions that initiate lateral roots. Together with the fact that *AHP6* is expressed at early stages of lateral root development, this gives a strong indication that AHP6 might act in a cell specific manner to inhibit cytokinin signaling during lateral root formation and that this could potentially happen in a similar manner to its role in the specification of vascular cell identity [8].

### AHP6 represses CK during LR initiation

It has been shown previously that exogenous CK treatments causes abnormal oblique/tangential pericycle cell divisions [5]. Consequently, we generated the hypothesis that the defective divisions during lateral root initiation in *ahp6* phenotype might be due to the lack of activity of a factor inhibiting CK signaling. To investigate this further, we quantified the relative frequency of stage I and stage II LR primordia that show abnormal pericycle cell divisions in WT with exogenous CK treatments (10 nM BAP (6-benzylaminopurine)) and compared this data with that generated for the *ahp6* mutant. Under these conditions, WT responded to CK by making oblique/tangential pericycle cell divisions as previously described [5] at both stage I and stage II of LR development (Figure 3c and 3d). These irregular cell divisions appeared to be of a similar nature to our previous observations of *ahp6*, although they occurred at a higher frequency. Furthermore, when treated with cytokinin, the two alleles of *ahp6* also showed an increase in the frequency at which the defective cell divisions occur (Figure 3c and 3d) (see also Table S1).

These results indicate that AHP6 most likely acts as a CK repressor during LR initiation. To confirm this, we analyzed *ahp6-1* harboring a CK catabolic enzyme (*CKX*, cytokinin oxidase) under the control of the *AHP6* promoter (*AHP6::CKX2*), reasoning that the defective phenotype would be rescued by lowering CK levels. We used two independent transgenic lines that had previously been shown to rescue the loss-of-protoxylem phenotype in *ahp6* [8] and did not observe any oblique/tangential pericycle cell divisions in stage I and II LR root primordia (Figure 2a and 2b - *ahp6*/*AHP6::CKX2*).

Taken together, our results show that AHP6 mediated CK inhibition plays a crucial role in the orientation of cell divisions during lateral root initiation.

### *AHP6* interacts with auxin transport during LR initiation

**Figure 4:**
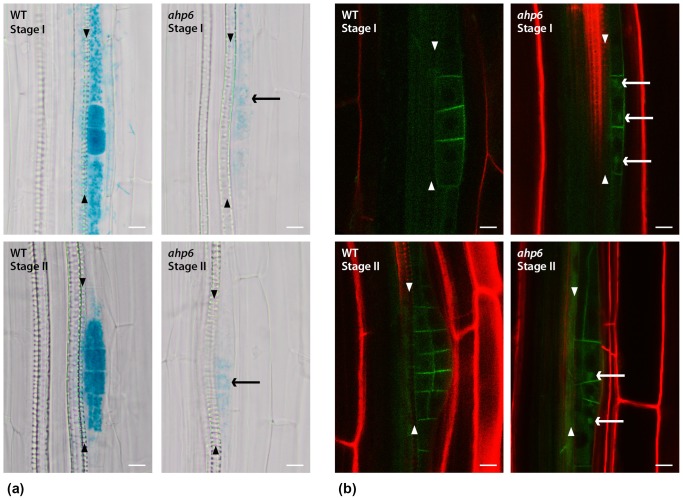
*AHP6* and its interaction with auxin. a) *DR5::GUS* signal is less intense in most of *ahp6* lateral root primordia. Additionally the auxin response pattern is sometimes altered, for example: in some stage I and stage II LR primordia auxin response could only be observed in approximately half the cells (arrows point the stained half). b) PIN1-GFP is localized at plasma-membrane in LR primordia of WT and *ahp6* mutant. Additionally, it shows an intracellular punctate pattern in around 35% *ahp6* LR primordia (n = 56) (arrows). Arrowheads: Xylem cell files. Bars: 10 µm.

It has been proposed that CK repression interferes with very early patterning events during the formation of LR, by disrupting the auxin maximum/gradient [5]. To address whether AHP6-mediated CK inhibition affects auxin distribution during lateral root initiation, we examined the expression of the auxin-responsive reporter, *DR5::GUS* in WT and *ahp6* during the first stages of lateral root development. Although there is some degree of variation in the intensity of the GUS signal within WT and in the *ahp6* mutant, we observed in most cases that the *DR5::GUS* staining was considerably weaker in *ahp6* lateral root primordia when compared to WT control plants (Figure 4a - compare WT with *ahp6*). We repeated this analysis using the fluorescent reporter *DR5rev::GFP* and observed the same effect with weaker signal in *ahp6* (Figure S2). In about 10% of primordia, we also observed deviations from the normal expression pattern of *DR5* (Figure 4a) suggesting a putative defect in auxin distribution.

Recently, a novel mode of CK action to modulate auxin activity has been uncovered in which CK regulates the endocytic recycling of the auxin efflux carrier PIN1 during lateral root development [6]. Consequently, we asked if *AHP6* could be involved in this type of auxin modulation. We analyzed the functional PIN1-GFP in WT and *ahp6-1* background. At stage I of WT lateral root development, PIN1 localizes predominantly on the anticlinal (transverse) sides of the pericycle founder cells ([2] and Figure 4b - WT stage I). At stage II, PIN1-GFP is also found at the periclinal (lateral) sides ([2] and Figure 4b - WT stage II). At stage I and II, we observed that the GFP signal exhibits an additional intracellular punctate pattern in *ahp6* LR primordia (Figure 4b - *ahp6* stage I and stage II) (see also Figure S3). These results resemble the original findings in which CK treatments were shown to have an effect on PIN1 localization by modulating its endocytic trafficking [6].

**Figure 5:**
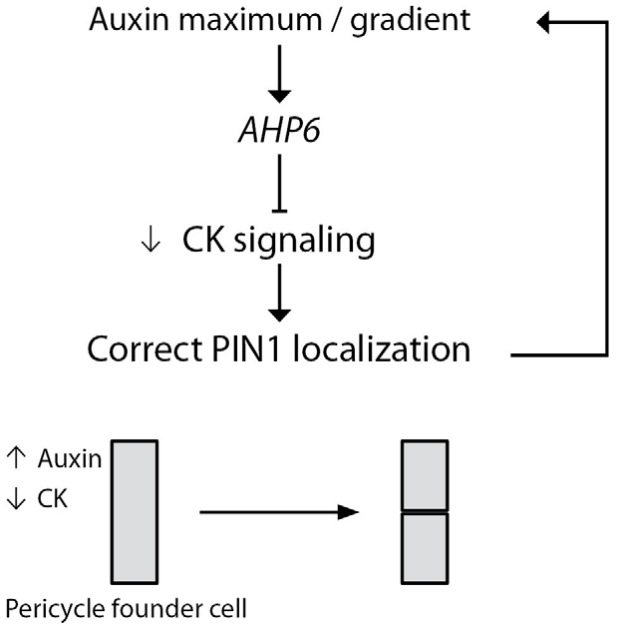
Model of *AHP6* action. In our model a domain of high auxin response promotes the expression of *AHP6*. AHP6 acts as an inhibitor of CK signaling to downregulate CK response in these cells. This decreased CK signaling affects the subcellular distribution of the auxin efflux carrier PIN1 which further refines the domain of high auxin response. We propose that this interaction between auxin and cytokinin via AHP6 is required to correctly orientate the plane of cell divisions in the lateral root primordia.

In conclusion, our data reveal that *AHP6* is a mediator of cytokinin inhibition during lateral root initiation and we propose that it may function through the modulation of PIN1 localization to generate the correct pattern of auxin response necessary for the patterning of lateral root primordia (Figure 5).

## Discussion

In the *Arabidopsis* root apical meristem, *AHP6* is expressed in the protoxylem cell files and the xylem-associated pericycle cells [8]. In this study, we show that *AHP6* is also expressed at different stages of lateral root (LR) development, including early stages of LR initiation. This specific expression of *AHP6* indicates that it may function in two distinct areas: firstly at the root meristematic zone, where it produces a positional signal in the xylem-associated pericycle cells to specify the competence for LR formation (priming the cell's fate); and secondly at the root differentiation zone, where it inhibits CK signaling in the pericycle founder cells to initiate lateral root formation. Although we cannot exclude the first hypothesis, our data strongly supports that *AHP6* mediates the inhibition of CK signaling to correctly orientate cell division in pericycle founder cells during lateral root initiation. The cell division phenotype of *ahp6* is very subtle. Firstly, as far as we observed the cell division defects are specific to lateral root primordia (although, we do not exclude the possibility that it may occur in embryos or in the upper parts of the plant in which *AHP6* is also expressed). Secondly, it presents low penetrance, i.e. the majority of *ahp6* stage I and II lateral root primordia do not have abnormal cell divisions. Finally, there are no major alterations in the number or time of emergence (data not shown) of LRs. Nonetheless, the aberrant cell division phenotype is consistent. The fact that there is no effect in the ultimate structure of the lateral root primordia could be due to the plasticity of plant development, in which plants have the ability to compensate aberrant cell divisions in order to correct the final LR pattern.

Under CK treatment conditions, WT lateral root primordia shows defects in the orientation of cell divisions resembling the *ahp6* abnormal cell division phenotype. These data are similar to previous findings showing a role for AHP6 in mediating protoxylem differentiation, where the *ahp6* mutation can be phenocopied by exogenous CK treatment [8]. In both cases exogenous CK treatment leads to a stronger phenotype and this could reflect the existence of additional (as yet unidentified) factors inhibiting cytokinin signaling. One slight difference between the two experiments is that a 10 nM CK treatment has a dramatic effect on enhancing the *ahp6-1* phenotype in the context of vascular development whereas in the context of regulating the orientation of pericycle cell divisions this effect is merely additive. This difference may reflect different sensitivities to CK for the different processes or it could be due to differences in the transport/accumulation of CK in the respective tissues. For example, it has been shown that a CK degrading enzyme, *AtCKX1*, is expressed in the pericycle around the lateral root branching points and when overexpressed *AtCKX1* as well as *AtCKX3* show defective lateral root phenotypes [14]. The expression of many *CKX* species is regulated by a variety of hormones including auxin and cytokinin [15]. Moreover, mutations in *ckx3* and *ckx5* have been shown to enhance the effect that *ahp6* has on regulating the size of the shoot apical meristem [12]. These raise the possibility that these CK oxidase genes also act synergistically with *AHP6* to regulate lateral root development.

Auxin is considered a morphogenetic trigger that specifies pericycle founder cells for lateral root initiation [16], [10]. In order to generate the correct pattern of auxin response proper localization of the PINs is needed [2]. In this work, we report defects in the subcellular localization of PIN1 and in the auxin signaling output (*DR5::GUS*) in *ahp6* mutants during lateral root initiation. However, the defects in the orientation of pericycle founder cell division are less frequent in *ahp6* mutants than those auxin-related defects. This could be because pericycle founder cells respond to certain thresholds of hormonal concentrations in order to achieve the correct orientation of cell division. Only when auxin/cytokinin levels are altered beyond a certain threshold will this result in defective orientations of cell division and the *DR5* marker may be not sensitive enough to report a range of auxin concentrations.

*AHP6* is targeted as a primary auxin response gene during vascular development and responds to auxin treatment in a similar manner to the primary auxin response gene *IAA2* [9]. We propose that auxin signaling would promote *AHP6* expression during pericycle cell specification. This might be achieved through the auxin signaling modules described to work upstream of the first pericycle founder cell divisions [17] and [18]. Future work will focus on the dependency of *AHP6* expression on those early auxin signaling modules. In turn, AHP6 represses CK signaling allowing correct PIN1 localization, and thus the formation of the auxin gradient which is required to pattern LR primordia. The model proposed (Figure 5) creates a feedback regulatory mechanism that integrates transcriptional and post- transcriptional levels of regulation. Feedback mechanisms are widely used during many development processes as they confer dynamics and robustness to biological systems [19].

In addition to vascular patterning and lateral root organogenesis, the interaction between cytokinin and auxin has been shown to regulate a large number of developmental processes, such as the formation of the embryonic root [20], root meristem size [21], vascular patterning [9] and the activity of the shoot apical meristem [22]. In this study, we have uncovered a new highly specific expression pattern during lateral root formation, and we have subsequently shown a specific role for AHP6 in inhibiting cytokinin signaling at this position. Given the broad expression pattern, and the importance of cytokinin signaling in diverse processes, we would predict that there will be many more reports showing a role for pseudo- histidine phosphotransfer proteins outside vascular development. Also, there is at least one pseudo- histidine phosphotransfer protein in the genomes of all higher plants which have been completely sequenced. Collectively these data suggest that the role of AHP6 in the inhibition of cytokinin signaling may be a frequently used component to regulate auxin-cytokinin crosstalk in higher plants.

## Materials and Methods

### Plant material

*Arabidopsis thaliana* plants, ecotype (Col-0) were used for all experiments, and all mutants and marker lines were in this background. The mutant lines *ahp6-1* and *ahp6-3* were previously described [8] as well as the transgenic lines *AHP6::GUS*, *AHP6::GFP* and *AHP6::CKX2* [8]. The *DR5::GUS* [23], *AUX1::AUX1-YFP* [24], *DR5rev::GFP* and *PINI::PIN1-GFP* [2] have also been previously described.

### Plant growth conditions and cytokinin treatment

Surface-sterilized seeds were stratified for 2 days at 4°C, in the dark, before plating onto 0.5 Murashige and Skoog medium with 1% sucrose and 0.4% phytagel. The plates were incubated at 22°C, 60% humidity and a cycle of 12 hr light/12 hr dark. For exogenous cytokinin treatments, seeds were germinated on medium containing 10 nM BAP.

### Data analysis of primary root and LRP development

To analyze root lengths, lateral root (LR) density, LR primordia density and LRP distribution, 10 days post germination (dpg) *Arabidopsis* roots were analysed from two to three independent experiments. LR density for WT and the two alleles of the *ahp6* mutant was determined by dividing the total number of emergent LRPs and LRs by the length of the LR branching zone [25]. LR primordia density was determined by dividing the total number of LRPs by the length of the lateral root- formation zone [25]. Lateral root-formation zone and lateral root - branching zone lengths were measured using the image-acquisition software cell∧B (Olympus). Data were statistically analysed using Excel 2007 (Microsoft) and XLSTAT 2012 (statistics package for Excel). Statistical significance (α<0.05) was determined using the non- parametric Wilcoxon-Mann-Whitney test.

The number of stage I and stage II LR primordia with defective cell divisions frequency was quantified by counting LR primordia with defective cell divisions at each stage and dividing it by the total number of primordia (n) at the respective stages in N roots (Table S1). The roots analyzed were from three independent experiments. In the case of roots treated with CK, two independent experiments were performed.

### Microscopy

Histochemical staining for GUS activity was performed as described [14]. Roots were mounted on glass microscope slides in an 8∶3∶1 solution of chloral hydrate: distilled water: glycerol [26]. Cleared roots were observed with an Olympus optical microscope (SZx1) using a 40× 0.75 objective and photographs were taken using an Olympus DP10 digital camera.

For DIC microscopy, cleared roots were observed in a Zeiss Axio Imager Z1 microscope with a Plan-Apochromat 63x/1.40 Oil objective and photographs were taken using Axiocam MR camera.

Confocal microscopy was performed using a laser scanning confocal microscope Leica SP2 (model SPS2 AOBS SE) with a HC PL APO CS 63x/1.30 glycerol objective. An Ar 488 nm laser was used for GFP and YFP excitation. Emission settings were 490–500 nm for GFP and 580–595 nm for YFP. Roots were transferred to microscope slides with propidium iodide to stain root cell walls (except for AUX1-YFP marker).

All experiments were performed in the 12 hr light period.

## Supporting Information

Figure S1 *ahp6* lateral root phenotype. a) Lateral root (LR) density for WT and the two alleles of the *ahp6* mutant. b) Lateral root primordia (LRP) density for WT and the two alleles of the *ahp6* mutant. c) Primary root growth for WT, *ahp6-1* and *ahp6-3*. From a to c, columns in bars display means and error bars are standard error of the mean (for Col-0 n = 13, *ahp6-1* n = 11, *ahp6-3* n = 8). Data is combined from two independent experiments. d) Lateral root primordia distribution (LRP) of WT and the two alleles of the *ahp6* mutant (for Col-0 n = 58, *ahp6-1* n = 84, *ahp6-3* n = 75). E - Emerged roots. Data is combined from three independent experiments. Statistical analysis was performed by pairwise comparisons of each parameter in the mutant alleles versus the same parameter in Col-0 using Wilcoxon-Mann-Whitney test (α<0.05).
(PDF)

Figure S2 *DR5::GFP* expression. *DR5::GFP* signal at initial stages of lateral root development in WT and *ahp6*. Red arrow: abnormal cell division. Bars: 10 µm.
(PDF)

Figure S3 PIN1-GFP localization. PIN1-GFP signal is located at the plasma-membrane in LR primordia and shows an additional intracellular punctate pattern in *ahp6* LR primordia (arrows). Arrowheads: Xylem cell files. Bars: 10 µm.
(PDF)

Table S1 Relative frequency of abnormal cell divisions at stage I and stage II of WT and *ahp6* lateral root (LR) primordia. Two growth conditions were analysed: with seeds that germinated in medium without cytokinins and with cytokinin (10 nM BAP). The raw data is displayed between brackets. n = number of primordia; N = number of roots.
(PDF)
