# Supplementary figures and images for: AHP6 Inhibits Cytokinin Signaling to Regulate the Orientation of Pericycle Cell Division during Lateral Root Initiation
Source: PLoS One. 2013 Feb 14;8(2):e56370. doi: 10.1371/journal.pone.0056370 (PMC3572949; doi:10.1371/journal.pone.0056370)

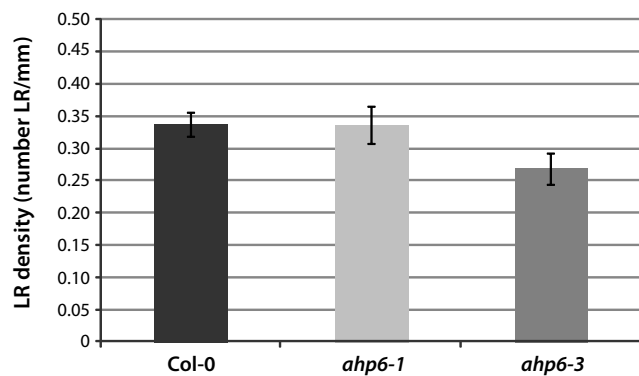

(a)

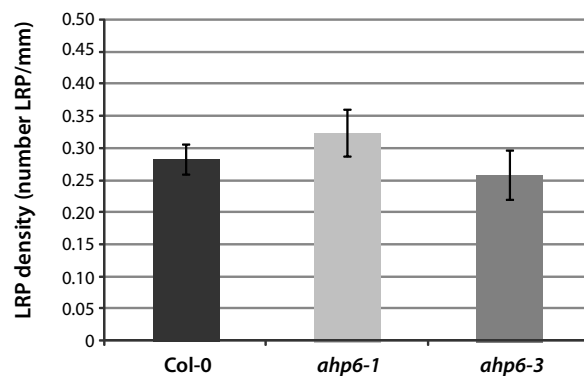

(b)

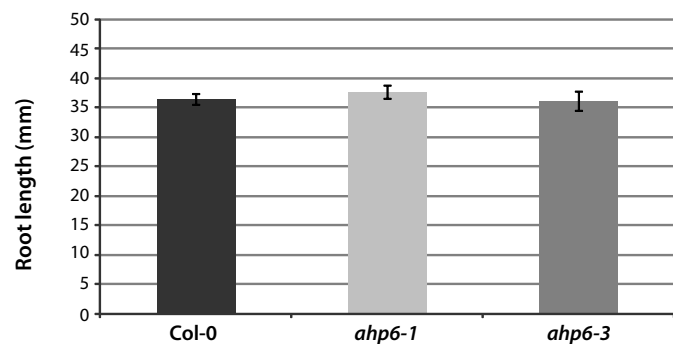

(c)

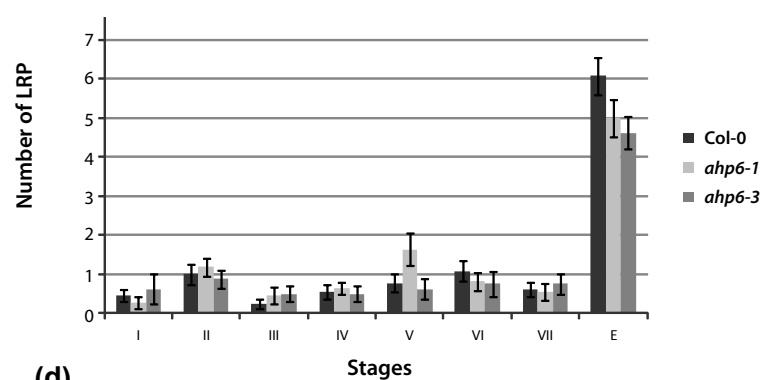

(d)

Supplement: Figure S1 — ahp6 lateral root phenotype. a) Lateral root (LR) density for WT and the two alleles of the ahp6 mutant. b) Lateral root primordia (LRP) density for WT and the two alleles of the ahp6 mutant. c) Primary root growth for WT, ahp6-1 and ahp6-3. From a to c, columns in bars display means and error bars are standard error of the mean (for Col-0 n = 13, ahp6-1 n = 11, ahp6-3 n = 8). Data is combined from two independent experiments. d) Lateral root primordia distribution (LRP) of WT and the two alleles of the ahp6 mutant (for Col-0 n = 58, ahp6-1 n = 84, ahp6-3 n = 75). E - Emerged roots. Data is combined from three independent experiments. Statistical analysis was performed by pairwise comparisons of each parameter in the mutant alleles versus the same parameter in Col-0 using Wilcoxon-Mann-Whitney test (α<0.05). (PDF) [file pone.0056370.s001.pdf]

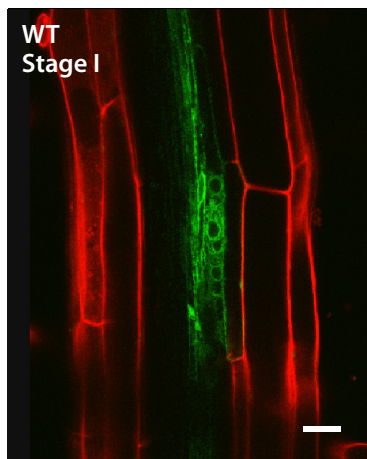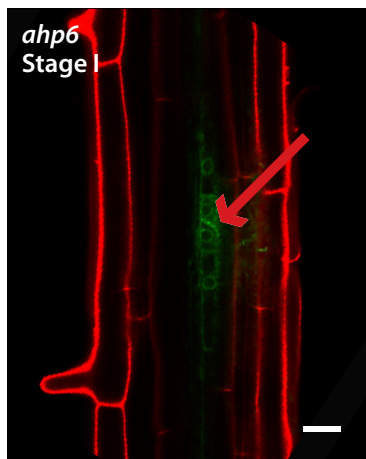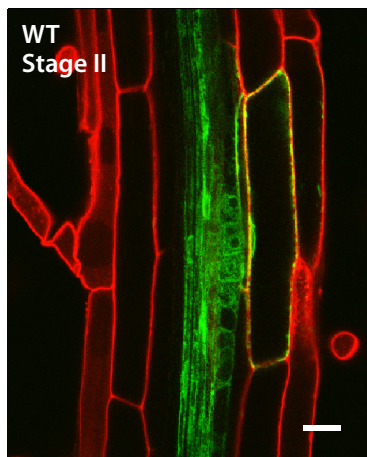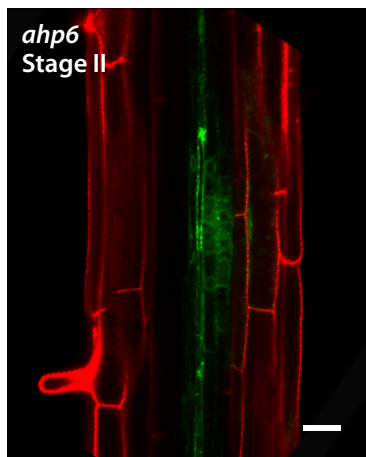

Supplement: Figure S2 — DR5::GFP expression. DR5::GFP signal at initial stages of lateral root development in WT and ahp6. Red arrow: abnormal cell division. Bars: 10 µm. (PDF) [file pone.0056370.s002.pdf]

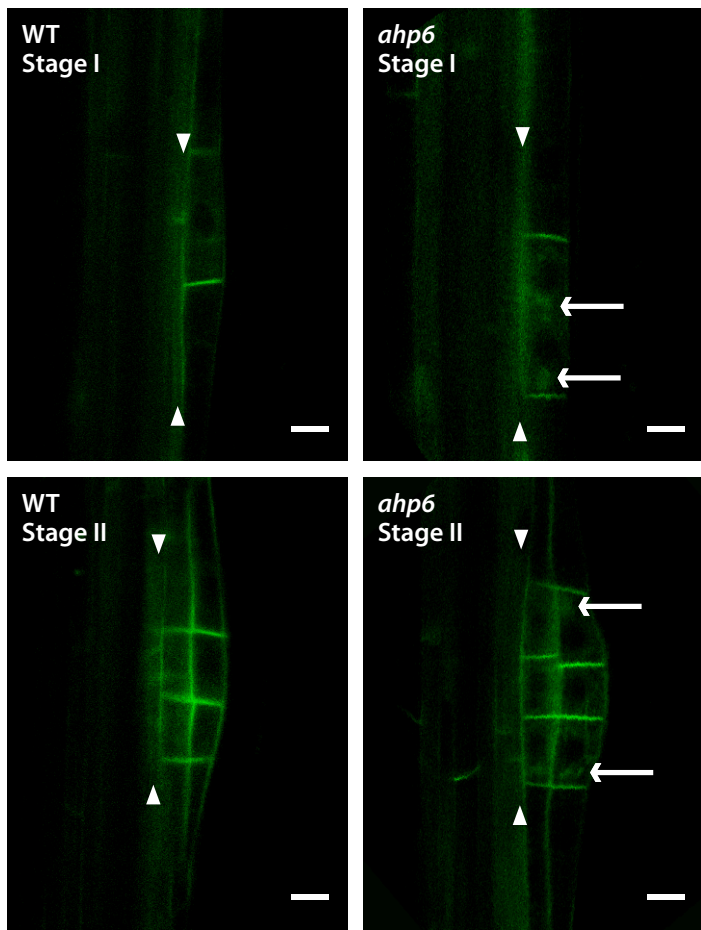

Supporting Information  
Figure S3

Supplement: Figure S3 — PIN1-GFP localization. PIN1-GFP signal is located at the plasma-membrane in LR primordia and shows an additional intracellular punctate pattern in ahp6 LR primordia (arrows). Arrowheads: Xylem cell files. Bars: 10 µm. (PDF) [file pone.0056370.s003.pdf]
