# Supplementary material for: AHP6 Inhibits Cytokinin Signaling to Regulate the Orientation of Pericycle Cell Division during Lateral Root Initiation
Source: PLoS One. 2013 Feb 14;8(2):e56370. doi: 10.1371/journal.pone.0056370 (PMC3572949; doi:10.1371/journal.pone.0056370)
Supplement: Table S1 — Relative frequency of abnormal cell divisions at stage I and stage II of WT and ahp6 lateral root (LR) primordia. Two growth conditions were analysed: with seeds that germinated in medium without cytokinins and with cytokinin (10 nM BAP). The raw data is displayed between brackets. n = number of primordia; N = number of roots. (PDF) [file pone.0056370.s004.pdf]

	Without exogenous CK			With exogenous CK(10nM BAP)	
	Stage I	Stage II		Stage I	Stage II
Col-0 (N=58)	0<br>(0/n=40)	0<br>(0/n=61)	Col-0 (N=51)	0.26<br>(9/n=35)	0.27<br>(13/n=48)
ahp6-1 (N=84)	0.24<br>(9/n=37)	0.06<br>(4/n=72)	*ahp6-1* (N=58)	0.41<br>(17/n=41)	0.50<br>(23/n=46)
ahp6-3 (N=75)	0.10<br>(5/n=48)	0.09<br>(5/n=55)	*ahp6-3* (N=56)	0.30<br>(11/n=37)	0.28<br>(15/n=53)

Supporting Information
Table S1
